# Prenatal Stress, Enteric Nervous System Development, and the Microbiota–Gut–Brain Axis: A Hypothesis-Generating Framework for Irritable Bowel Syndrome and Fibromyalgia

**DOI:** 10.3390/ijms27167177

**Published:** 2026-08-11

**Authors:** Noemi Császár-Nagy, István Bókkon

**Affiliations:** 1 Faculty of Public Governance and International Studies, Ludovika University of Public Services, H-1083 Budapest, Hungary; csaszar-nagy.noemi.andrea@uni-nke.hu; 2 Psychosomatic Outpatient Clinics, H-1037 Budapest, Hungary; 3 Neuroscience and Consciousness Research Department, Vision Research Institute, Inc., Lowell, MA 01854, USA

**Keywords:** maternal mental stress, enteric nervous system microbiota–gut–brain axis, long-term epigenetic implicit memories, irritable bowel syndrome, fibromyalgia

## Abstract

The enteric nervous system (ENS) can function semi-autonomously from the central nervous system (CNS) in regulating complex gastrointestinal processes and exhibits substantial developmental, epigenetic, neuroimmune, and adaptive plasticity. We propose the concept of Stress-Induced Long-term Epigenetic Implicit Memory (SLEIM) as a hypothesis-generating theoretical framework suggesting that prenatal maternal stress may contribute to persistent biological alterations within ENS-related pathways through interacting epigenetic, neuroimmune, neuronal, glial, and microbiota-associated mechanisms. The precise biological substrates and mechanisms underlying this proposed framework remain unknown. Through the microbiota–gut–brain axis (MGBA), such stress-related biological alterations may influence physiological communication between the ENS and CNS and in turn affect stress-response systems, including HPA axis activity, immune signalling, cortisol regulation, mast-cell activation, and cytokine balance. The frequent comorbidity of fibromyalgia (FM) and irritable bowel syndrome (IBS) suggests the existence of shared pathogenic mechanisms involving central sensitisation, neuroimmune processes, and MGBA dysfunction. In this study, we therefore also address dependency-related characteristics and autonomy vulnerabilities reported in some patients with FM and consider how developmental, psychological, neurobiological, and illness-related factors may contribute to these patterns. Within the proposed SLEIM framework, prenatal stress-related biological influences may represent a potential developmental pathway that contributes to vulnerability to IBS, FM, and related functional disorders later in life. However, these relationships remain hypothetical and require future empirical investigation.

## 1. Introduction

Prenatal stress is increasingly recognised as an important biological factor capable of influencing multiple aspects of foetal development through interacting neuroendocrine, immune, metabolic, and epigenetic pathways. Maternal stress, anxiety, and depression, along with other adverse psychosocial conditions during pregnancy, may affect foetal physiology through glucocorticoids, inflammatory mediators, neuroimmune signalling, and alterations in the maternal microbial composition. These prenatal influences have been associated with long-term effects on neurodevelopment, stress regulation, immune function [1], and vulnerability to a range of chronic disorders later in life [2].

The enteric nervous system, the central nervous system, and the immune system develop in parallel [3,4] during foetal life and interact extensively through bidirectional biological pathways [5,6,7]. Increasing evidence suggests that prenatal environmental influences affect these systems through epigenetic regulation, neuroimmune mechanisms, alterations in microbial metabolites, and changes in stress-response pathways [8,9,10]. Within this context, the MGBA has emerged as an important framework for understanding communication between the gastrointestinal tract, immune system, and CNS [11,12,13].

In our previous work, we proposed the concept of Stress-Induced Long-term Epigenetic Implicit Memory (SLEIM) as a hypothesis-generating theoretical framework [1,2], which suggests that prenatal stress-related biological influences [8] may become persistently embedded within ENS-associated pathways through interacting epigenetic, neuroimmune, neuronal, glial, and microbiota-related processes [14]. However, the precise biological substrates, encoding mechanisms, persistence, retrieval processes, and measurable correlates of these proposed alterations remain unknown. Accordingly, SLEIM should be regarded as a theoretical construct intended to generate testable hypotheses rather than an established biological mechanism.

Within our proposed framework, prenatal stress-related biological influences may contribute to long-term alterations in physiological responsiveness through interactions between the ENS, CNS, immune system, and microbiota [8]. Such alterations could influence stress regulation, neuroimmune signalling, intestinal function, visceral sensitivity, and pain-related processes. Nevertheless, direct evidence demonstrating the existence of SLEIM as a distinct biological entity is currently lacking, and the framework requires substantial empirical validation.

Several biological pathways may plausibly contribute to the processes proposed above, including epigenetic regulation through DNA methylation, histone modifications, and non-coding RNAs [14]; neuroimmune signalling involving cytokines and inflammatory mediators; microbial metabolites such as short-chain fatty acids (SCFAs) and tryptophan-derived compounds [11]; autonomic pathways, particularly vagal signalling [13]; and endocrine mechanisms associated with HPA axis activity. Together, these pathways may contribute to long-term biological adaptations within the MGBA and therefore warrant further investigation.

Fibromyalgia (FM) and irritable bowel syndrome (IBS) frequently coexist [15] and share several biological and clinical features [16], including altered pain processing, central sensitisation [17], neuroimmune dysregulation, disturbances within the MGBA [15], and increased psychological burden [18]. These observations raise the possibility that common developmental and regulatory mechanisms may contribute to vulnerability across both conditions [15].

**Figure 1:**
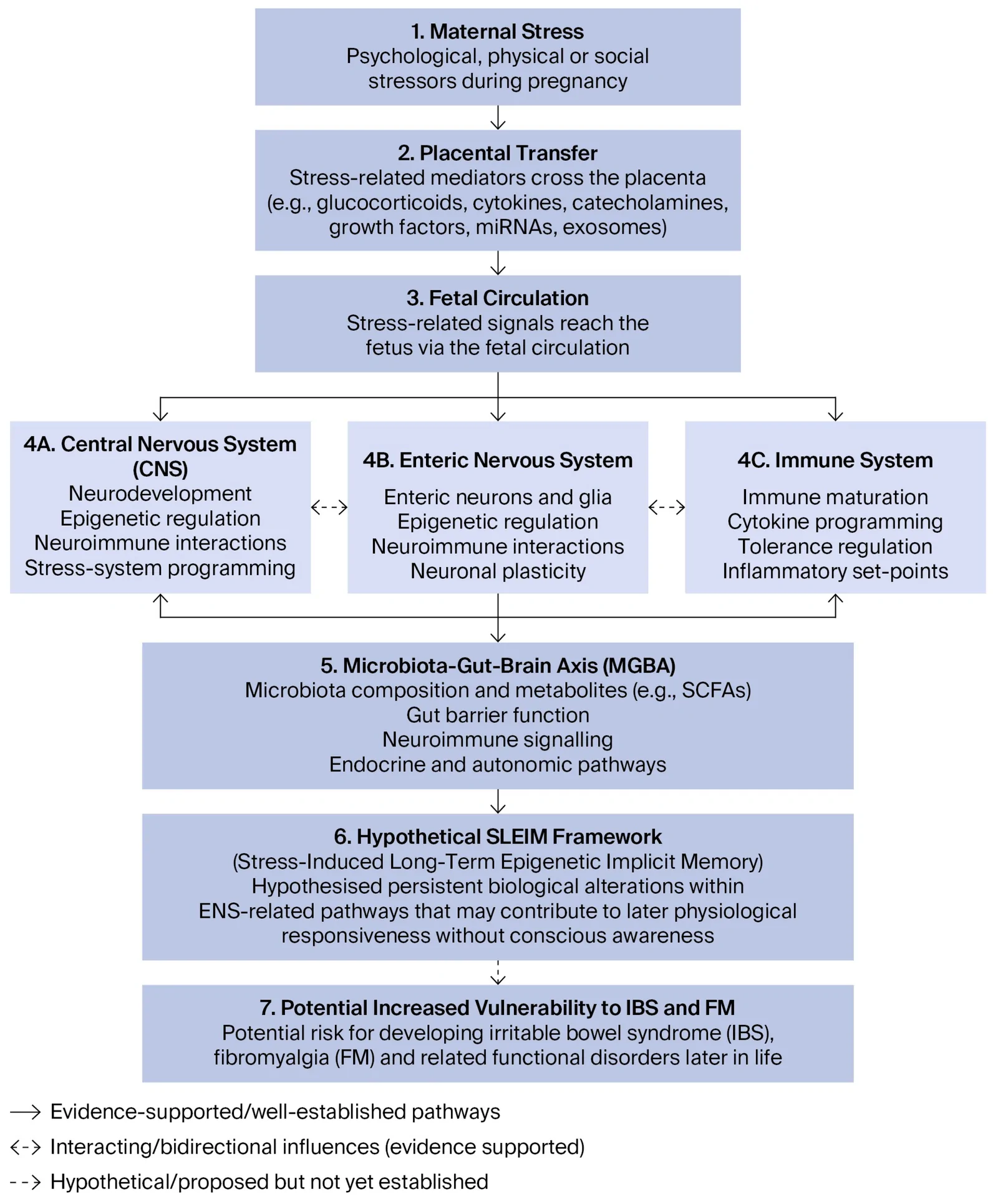
Hypothesised developmental pathways linking prenatal stress, the Microbiota-Gut–Brain-Axis, and long-term vulnerability to IBS and FM within the Stress-Induced Long-term Epigenetic Implicit Memory framework.

In this hypothesis-driven narrative review, we explore whether prenatal stress-related biological influences affecting ENS-associated pathways could represent one potential developmental factor contributing to later vulnerability to IBS and FM. We further discuss dependency-related characteristics and autonomy-related vulnerabilities that have been described in some patients with chronic pain conditions and consider how developmental, psychological, neurobiological, and illness-related factors may interact within this broader framework [18]. The proposed conceptual relationships between prenatal stress, foetal developmental systems, the MGBA, the hypothetical SLEIM framework, and potential vulnerability to IBS and FM are summarised in Figure 1, which presents an original conceptual model developed by the authors based on the literature reviewed in the present article [1,2].

## 2. The Enteric Nervous System, the Microbiota–Gut–Brain Axis, and Long-Term Biological Adaptation

The ENS is a highly complex intrinsic neural network of the gastrointestinal tract that plays a central role in regulating gastrointestinal motility, secretion, vascular function, barrier integrity, immune interactions, and host–microbiota communication [3,5,6]. Through extensive interactions with the CNS, immune system, endocrine pathways, and gut microbiota, the ENS contributes to the maintenance of physiological homeostasis [6] and adaptive responses to environmental challenges [19].

From an evolutionary perspective, enteric neural regulation represents one of the most ancient forms of nervous system organisation [4]. Comparative and evolutionary studies have suggested that several fundamental principles of enteric neural control emerged early during animal evolution and may have preceded the development of more complex centralised nervous systems in some species [3]. However, in mammalian embryonic development, the ENS and CNS are closely interconnected developmental systems that arise through coordinated neurodevelopmental processes rather than as entirely independent structures [5].

Within the developing foetus, neural crest-derived progenitor cells contribute to ENS formation, while the ENS, CNS, and immune system undergo parallel maturation influenced by interacting genetic, epigenetic, neuroimmune, endocrine, microbial, and environmental factors. Consequently, prenatal influences may affect multiple developing systems simultaneously through interconnected developmental pathways.

The ENS and the CNS communicate bidirectionally through the MGBA, a complex communication network linking the gastrointestinal tract, gut microbiota, immune system, autonomic nervous system (ANS), neuroendocrine pathways, ENS, and CNS [13,19]. The gut microbiota comprises bacteria, viruses, fungi, and archaea together with their diverse metabolites, many of which participate in host physiological regulation [11]. Within the ANS, the vagus nerve represents a major pathway of bidirectional gut–brain communication, whereas the HPA axis functions as a principal neuroendocrine stress-response system coordinating adaptive responses to environmental and internal stressors [8,13].

Although the ENS possesses substantial intrinsic regulatory capacity and can generate complex patterns of gastrointestinal activity independently of direct CNS control [7,20], physiological gut function normally depends on continuous bidirectional communication among the ENS, CNS, immune system, and microbiota [12,13,19]. The ENS and CNS share numerous structural, cellular, molecular, and neurochemical characteristics, including the presence of many common neurotransmitters, neuronal signalling pathways, and glial cell functions [5,20,21]. Furthermore, several developmental mechanisms involved in CNS formation also contribute to ENS ontogeny [14,21,22,23].

Notably, approximately 90% of vagal fibres are afferent, indicating that gut-derived information continuously influences central neural processing and that brain–gut communication is fundamentally bidirectional [13]. This observation highlights the importance of considering the gastrointestinal system not merely as a target of central regulation but also as an active contributor to neurophysiological regulation.

Epigenetic regulation has been proposed as an important mechanism underlying long-lasting biological adaptation [24]. DNA methylation, histone modifications, and non-coding RNAs, including microRNAs, are known to influence neuronal development, cellular differentiation, stress responsiveness, and long-term regulation of gene expression [22,24,25]. Similarly, ENS development from neural crest-derived progenitor cells is regulated by interacting genetic, epigenetic, neuroimmune, and signalling pathways [14,23,26]. Epigenetic regulation therefore represents a plausible mechanism through which prenatal environmental influences may exert persistent effects on ENS-related developmental processes [8,14,22].

Experimental and clinical studies further suggest that the ENS exhibits substantial adaptive plasticity [27]. Long-lasting physiological responses following inflammation, microbial alterations, nutritional exposures, or stress-related stimuli indicate that prior biological experiences may influence subsequent ENS function [28,29]. Although such observations demonstrate persistent physiological adaptation and plasticity within ENS-associated pathways, they should not be interpreted as evidence for cognitive memory processes analogous to those of the CNS. Rather, they provide a conceptual basis for considering whether long-term biological influences may become embedded within ENS-related regulatory systems and contribute to later physiological responsiveness.

Within this context, the proposed SLEIM framework is not intended to imply the existence of a CNS-like memory system within the ENS. Instead, it serves as a hypothesis-generating theoretical construct suggesting that prenatal stress-related biological influences may become persistently embedded within ENS-related pathways through interacting epigenetic, neuroimmune, neuronal, glial, and microbiota-associated mechanisms. The precise biological substrates, persistence mechanisms, and measurable correlates of these proposed processes remain unknown and require future empirical investigation.

## 3. Maternal Stress in Pregnant Women

Various biological, environmental, and psychosocial factors influence the composition of the gut microbiota and the MGBA during pregnancy and may affect foetal development and long-term health outcomes [30]. These factors include maternal nutrition, obesity, infections, medications (including antibiotics and antidepressants), environmental toxins [31], smoking [32], alcohol consumption [33], diet-induced dysbiosis [34], psychological stress [35], genetic and epigenetic influences, and maternal age. Prenatal maternal stress responses [8] are complex, multifactorial, and highly variable among individuals [36]; accumulating evidence suggests that such influences may contribute to developmental programming processes with potential long-term consequences for offspring health.

Maternal psychological stress, anxiety, and depression during pregnancy affect a substantial proportion of pregnant women worldwide [37,38]. Increasing evidence suggests that prenatal stress exposure may be an important developmental influence that affects foetal physiology through interactions between neuroendocrine, immune, epigenetic, metabolic, and neurodevelopmental pathways [8,36,39]. It has also been suggested that prenatal stress exposure [9] may contribute to long-term alterations in stress regulation, neurodevelopment, immune function, and vulnerability to various physical and mental health conditions later in life [40,41].

Pregnancy-related physiological and psychosocial changes may increase vulnerability to stress, anxiety, and depressive symptoms [37,38,42]. Epidemiological studies indicate that depressive symptoms occur in a substantial proportion of pregnant women, with a considerable subset meeting the criteria for major depressive disorder during pregnancy [37,43]. Kiyak et al. [42] reported that symptoms of stress, anxiety, and depression were highly prevalent among pregnant women and were associated with coping style. In particular, emotion-focused coping was positively associated with psychological distress, whereas problem-focused coping showed a negative association.

Other studies have similarly demonstrated that elevated maternal stress during pregnancy is associated with poorer obstetric outcomes and adverse maternal mental health outcomes [44,45]. Anxiety symptoms during pregnancy are also common and may persist across successive pregnancies [37,38]. A comprehensive analysis of systematic reviews conducted by Al-Abri et al. [37] demonstrated substantial prevalence rates for antenatal, perinatal, and postnatal depression worldwide. Likewise, anxiety disorders affect a significant proportion of pregnant women and appear to be more common [38] than previously recognised.

Thongsomboon et al. [46] reported that perceived stress symptoms were significantly associated with marital conflict, family conflict, separation from a partner, and exposure to physical or psychological trauma. Additional risk factors for maternal depression and anxiety during pregnancy include limited social support, social isolation, unintended pregnancy, multiparity, maternal age, marital difficulties [47], and a personal or family history of affective disorders [48].

Furthermore, pregnancy-related stress is highly prevalent, with some studies suggesting that a large majority of pregnant women experience varying degrees of psychological stress during gestation [49]. Consequently, early identification and monitoring of maternal mental health remain important clinical priorities [8]. Recent findings have further highlighted the importance of recognising risk factors associated with antenatal depression and improving early detection during pregnancy [50].

One proposed mechanism through which maternal prenatal stress may influence long-term developmental outcomes involves alterations in foetal neurodevelopment and biological programming processes [51]. Maternal prenatal stress is frequently associated with alterations in cortisol regulation, stress-related neuroendocrine activity, inflammatory signalling, and neurotransmitter systems, all of which may influence foetal brain development [8]. The HPA axis represents a central component of the stress-response system, while glucocorticoid-mediated signalling has been implicated in developmental programming processes during pregnancy [8,10].

Elevated glucocorticoid exposure has been associated with alterations in the development of foetal brain regions involved in stress regulation, including the hippocampus and amygdala [52,53]. Such developmental influences may affect later cognitive functioning, emotional regulation, and stress responsiveness. Neuroimaging studies have reported altered neural connectivity patterns and reduced hippocampal volumes in infants exposed to elevated prenatal maternal anxiety [52]. At the molecular level, chronic prenatal stress has been associated with alterations in DNA methylation patterns involving glucocorticoid receptor-related genes, including NR3C1, which may contribute to long-term changes in stress responsivity and vulnerability to affective disorders [10].

Importantly, prenatal stress-related biological influences are unlikely to affect a single developmental system in isolation [8,9,53]. Rather, current evidence suggests that the CNS, ENS, immune system, and microbiota-associated pathways undergo parallel and interacting developmental processes during foetal life [54,55]. Consequently, prenatal stress may contribute to coordinated biological alterations across multiple systems, providing a potential framework for understanding later vulnerability to disorders involving gut–brain interactions [13], stress regulation, chronic pain, immune dysregulation, IBS, and fibromyalgia [18,56,57,58]. Within the proposed SLEIM framework, such influences are considered hypothetical contributors to long-term biological adaptation rather than evidence of a specific established pathogenic mechanism [18,56,57,59].

### Maternal Stress During Pregnancy, Foetal Development, and Preventive Considerations

Given the growing evidence linking maternal stress exposure to foetal developmental outcomes, interventions aimed at reducing psychological distress during pregnancy have attracted increasing attention [8,60]. Approaches such as mindfulness-based interventions [61,62,63], cognitive behavioural therapy, relaxation techniques, meditation, prenatal yoga, hypnosis, sleep optimisation [64,65,66], regular physical activity, healthy dietary habits, and broader lifestyle modifications have been investigated as potential strategies for improving maternal psychological well-being during pregnancy [67,68,69].

In addition, social and emotional support from partners, family members, healthcare professionals, and community networks appears to play an important role in maternal psychological adjustment during pregnancy [37,48]. Early identification and management of clinically significant symptoms of stress, anxiety, and depression may contribute to improved maternal well-being and potentially more favourable pregnancy outcomes [37,47]. Consequently, routine assessment of maternal mental health is increasingly being recognised as an important component of comprehensive prenatal care [38,47].

## 4. Irritable Bowel Syndrome, Psychological Distress, and the Microbiota–Gut–Brain Axis

Irritable bowel syndrome (IBS) is a common disorder of gut–brain interaction characterised by recurrent abdominal pain associated with altered bowel habits, including constipation, diarrhoea, or mixed symptom patterns [70]. According to contemporary epidemiological studies, IBS is one of the most prevalent functional gastrointestinal disorders, affecting a substantial proportion of the global population [70,71].

The pathophysiology of IBS is complex and multifactorial. Proposed contributing mechanisms include genetic and epigenetic influences, immune dysregulation, altered stress-response systems, HPA axis dysfunction, disturbances within the MGBA [1,22], dysbiosis, altered gastrointestinal motility, visceral hypersensitivity, post-infectious changes, food-related sensitivities, carbohydrate malabsorption, and alterations in serotonergic signalling [72,73]. Despite considerable research efforts, no single mechanism fully explains the development of IBS, and its biological basis remains incompletely understood [70,71].

Over the past two decades, the interactions between psychological factors and IBS have been more closely examined. Individuals with IBS frequently report higher levels of anxiety, depression, perceived stress, and psychological distress compared with healthy controls [74,75,76]. These psychological factors appear to interact bidirectionally with gastrointestinal symptoms through neural, endocrine, immune, and microbiota-mediated pathways [12,19]. Consequently, emotional distress may influence symptom severity, while persistent gastrointestinal symptoms may further contribute to psychological burden [76].

Women with IBS appear particularly vulnerable to this reciprocal interaction. Studies have reported increased levels of psychological distress, enhanced visceral sensitivity, altered gastrointestinal motility, and differences in microbiota composition among women with IBS [71,73,74,77]. These biological and psychological factors may interact within the MGBA, potentially contributing to symptom persistence and fluctuations over time.

Growing evidence suggests that the gut microbiota influences ENS activity, immune signalling, intestinal barrier function, and afferent communication with the CNS [12,77,78]. Dysregulation of these interconnected systems is increasingly being recognised as an important component of IBS pathophysiology [71,73,77]; altered MGBA signalling may contribute not only to gastrointestinal dysfunction but also to changes in pain perception, stress responsiveness, and emotional regulation [12,77,78].

Psychological comorbidities are also frequently reported among pregnant and postpartum women with IBS [74,79]. Although the direction of causality remains uncertain, these observations support the view that IBS, psychological distress, and MGBA dysregulation are closely interconnected phenomena [12,76,77]. Within the broader conceptual framework proposed in this review, prenatal stress-related influences on developing CNS-, ENS-, immune-, and microbiota-associated pathways may represent one potential factor contributing to later vulnerability to IBS [1,2]. However, such developmental pathways remain hypothetical and require further empirical investigation.

## 5. Fibromyalgia

Fibromyalgia (FM) is a chronic pain syndrome characterised by widespread musculoskeletal pain accompanied by a constellation of symptoms that commonly include fatigue, sleep disturbances, cognitive dysfunction, anxiety, depression, and a variety of somatic complaints [80,81]. Gastrointestinal symptoms, particularly irritable bowel syndrome (IBS), are also highly prevalent among individuals with FM [15,82,83]. FM predominantly affects women and is increasingly recognised as a complex multisystem condition involving interactions between neurobiological, immunological, endocrine, genetic, environmental, and psychosocial factors [18,84].

Evidence suggests that FM has a substantial heritable component, with prevalence estimates in the general population ranging from approximately 0.5% to 12% depending on the diagnostic criteria and population studied [85,86,87,88]. Familial aggregation studies have reported a markedly increased risk among first-degree relatives of affected individuals [89]. Although the precise mode of inheritance remains unclear, current evidence supports a polygenic and multifactorial model involving interactions between genetic susceptibility and environmental influences [86,88].

The association between FM and gastrointestinal dysfunction has been repeatedly documented [15,82,83]: Triadafilopoulos et al. reported bowel dysfunction in a substantial proportion of patients with FM [90], while more recently, Erdrich et al. confirmed that IBS is one of the most common comorbid conditions in FM and noted that mixed and constipation-predominant IBS subtypes appear particularly frequent [82]. These observations further support the concept of shared biological pathways linking chronic pain conditions and disorders of gut–brain interaction [15,82,83].

Emerging evidence has also implicated the gut microbiota in FM pathophysiology. In a germ-free mouse model, Cai et al. demonstrated that transplantation of faecal microbiota from patients with FM induced behavioural, immunological, and metabolomic alterations that resembled several features observed in FM [91]. Notably, these effects were not observed following transplantation from healthy controls, and the replacement of FM-associated microbiota with that derived from healthy donors was associated with a reduction in pain-related behaviours. Although these findings do not establish causality in humans, they provide further evidence supporting a potential role of microbiota-associated mechanisms in FM and highlight the importance of the MGBA as an area for future investigation [15,91].

Despite extensive research, the pathophysiology of FM remains incompletely understood. Multiple interacting mechanisms have been proposed, including genetic susceptibility, environmental influences, physical and emotional trauma, infections, neuroimmune alterations, endocrine dysregulation, and abnormalities in central pain processing [84]. Of these mechanisms, central sensitisation is currently regarded as one of the most important and consistently supported explanatory frameworks [92]. According to this model, altered CNS processing contributes to amplification of sensory input, enhanced pain perception, and increased responsiveness to both painful and non-painful stimuli [93].

Importantly, the coexistence of FM and IBS, together with evidence implicating microbiota-associated pathways, neuroimmune interactions, stress-response systems, and altered pain processing, suggests that these conditions may share partially overlapping biological mechanisms [15,82,84,91]. However, the precise nature of these relationships remains incompletely understood. Within the broader conceptual framework proposed in this review, prenatal stress-related influences affecting developing CNS-, ENS-, immune-, and microbiota-associated pathways may be a potential contributor to later vulnerability [1,2,94]. Nevertheless, FM should be regarded as a multifactorial condition for which no single explanatory model is currently sufficient [18,84].

## 6. The Relationship Between Irritable Bowel Syndrome and Fibromyalgia

The frequent co-occurrence of irritable bowel syndrome (IBS) and fibromyalgia (FM) has prompted increasing interest in the possibility that these disorders may share partially overlapping biological mechanisms [15,16]. Proposed areas of overlap include altered pain processing, central sensitisation, neuroimmune interactions, stress-response dysregulation, microbiota-related mechanisms, and disturbances within the MGBA [15,84,91].

Several investigators have suggested that IBS and FM may represent different clinical manifestations within a broader spectrum of disorders involving gut–brain interaction and altered sensory processing. Berstad et al. followed more than 1100 patients with IBS over a nine-year period and proposed that gastrointestinal symptoms may precede the later emergence of systemic symptom clusters, including chronic widespread pain and myalgic encephalomyelitis/chronic fatigue syndrome-like presentations [95]. However, the temporal association between these conditions does not establish a direct causal relationship, and the mechanisms linking IBS and FM remain incompletely understood.

Experimental studies have provided additional evidence supporting a role for microbiota-associated mechanisms. De Palma et al. transplanted faecal microbiota obtained from patients with diarrhoea-predominant IBS (IBS-D), with or without anxiety, into germ-free mice and observed alterations in gastrointestinal function, low-grade inflammatory responses, and anxiety-like behaviours [96]. These findings suggest that microbiota-associated factors may influence both gastrointestinal and behavioural phenotypes through interconnected biological pathways.

Similarly, Lin et al. reported that faecal microbiota transplantation from healthy donors was associated with improvements in anxiety and depressive symptoms among patients with IBS-D [97]. Although the underlying mechanisms remain under investigation, such findings further support the relevance of microbiota–gut–brain interactions in disorders characterised by both gastrointestinal and psychological symptoms.

Garofalo et al. proposed that dysbiosis may represent one of several upstream biological factors contributing to symptom development in both IBS and FM [15]. Emerging evidence also suggests potential roles for neuroimmune signalling, altered barrier function, microbial metabolites, epigenetic regulation, and stress-related physiological adaptations [98]. However, the relative contribution of each mechanism remains uncertain, and it is unlikely that a single biological pathway fully explains the development of either condition.

As summarised in Table 1, IBS and FM demonstrate substantial overlap across clinical, neurobiological, psychological, microbiota-associated, and stress-related domains. These converging observations support the possibility that partially shared biological mechanisms contribute to the frequent co-occurrence of the two conditions, although the precise nature of these relationships remains incompletely understood.

Within the conceptual framework proposed in this review, developmental influences affecting CNS-, ENS-, immune-, and microbiota-associated pathways may be a potential factor contributing to later vulnerability. Nevertheless, IBS and FM should be regarded as multifactorial conditions arising through complex interactions among biological, psychological, environmental, and developmental influences, rather than through a single pathogenic mechanism.

## 7. Dependency-Related Characteristics and Autonomy Vulnerabilities in Fibromyalgia

Fibromyalgia is increasingly recognised as a complex biopsychosocial condition that extends beyond chronic pain and somatic symptom burden. In addition to widespread pain, fatigue, sleep disturbance, cognitive complaints, and gastrointestinal symptoms, many patients experience difficulties in emotional regulation, stress adaptation, self-management, and everyday functioning [103,104,105]. Importantly, these characteristics should not be interpreted as evidence of personality pathology or developmental deficit. Rather, they may reflect the cumulative consequences of chronic pain, prolonged functional impairment, repeated healthcare experiences, uncertainty regarding diagnosis, social invalidation, and persistent physiological stress [104]. Such processes may gradually influence coping behaviour, self-efficacy, autonomy, and patterns of adaptation to illness. Within the chronic pain literature, reduced self-efficacy, passive coping strategies, pain catastrophising, avoidance behaviour, heightened bodily vigilance, and increased reliance on external sources of support have been repeatedly reported among individuals with fibromyalgia and related chronic pain conditions [105,106,107]. These features are not unique to fibromyalgia; however, they may contribute substantially to symptom persistence, disability, and reduced quality of life.

### 7.1. Chronic Pain, Coping, and Self-Regulation

Chronic pain is a multidimensional phenomenon influenced by interacting biological, psychological, and social processes rather than by nociceptive mechanisms alone [103,104]. Contemporary biopsychosocial models conceptualise chronic pain as the outcome of dynamic interactions among neurobiological processes, emotional regulation, cognitive appraisal, behavioural adaptation, social context, and environmental influences [103,104].

Within this framework, coping refers to the cognitive, emotional, and behavioural strategies individuals use to manage stressors, symptoms, and perceived threats [105,106,107]. Research has demonstrated that patients with fibromyalgia frequently exhibit lower levels of self-efficacy, greater use of passive coping strategies, increased pain catastrophising, heightened bodily vigilance, stress sensitivity, sleep disturbances, autonomic dysregulation, and elevated emotional distress compared with healthy controls [105]. Gastrointestinal symptoms and comorbid irritable bowel syndrome are also commonly reported [15,82]. Such patterns may influence symptom perception, illness behaviour, treatment engagement, and long-term adaptation to chronic illness [103,104]. Consequently, understanding coping and self-regulatory processes has become an important component of contemporary fibromyalgia research [105].

Current evidence supporting these observations is relatively strong and is reflected in recent reviews and meta-analyses, including work by Clauw [108], Siracusa and colleagues, Bellato and colleagues, Häuser and collaborators [18], and more recent reviews by Galvez-Sánchez and others focusing on cognitive functioning, stress regulation, autonomic processes, and neuropsychological aspects of fibromyalgia [92,105].

### 7.2. Premorbid Vulnerabilities and Stress-Related Risk Factors

A growing body of literature suggests that some individuals with fibromyalgia may exhibit premorbid vulnerabilities associated with later symptom development [18,108]. However, the available evidence primarily demonstrates associations rather than causal relationships. Factors that have been linked to increased fibromyalgia risk include adverse childhood experiences, early-life stress exposure, anxiety-related traits, sleep disturbances, autonomic dysregulation [98], and pre-existing functional gastrointestinal disorders such as irritable bowel syndrome [15,82].

Several studies have contributed to this literature, including investigations of familial aggregation, central pain processing, biopsychosocial vulnerability, and stress-related mechanisms in fibromyalgia [89,108]. Nevertheless, these findings should be interpreted cautiously, as they do not establish direct premorbid causation. Rather, they suggest that certain biological, psychological, and environmental factors [18] may increase vulnerability to chronic pain and stress-related disorders across the lifespan.

### 7.3. Developmental and Prenatal Perspectives

The developmental and prenatal dimensions of fibromyalgia remain incompletely understood and have received considerably less direct investigation than other proposed pathophysiological mechanisms. Concepts such as prenatal stress exposure, altered maternal–foetal signalling, disturbances in ENS–CNS developmental interactions, and early microbiome-related influences are not currently supported by fibromyalgia-specific causal evidence [8,51]. Instead, these ideas derive largely from broader research fields, including the developmental origins of health and disease (DOHaD) framework [56], prenatal stress research, neurodevelopmental biology, ENS development [53], and microbiome science [53].

Within this context, prenatal and developmental influences may be viewed as factors that are consistent with, compatible with, or potentially contributory to later stress vulnerability and chronic illness susceptibility [56,59,109,110]. However, they should be regarded as hypothesis-generating concepts rather than established explanations for fibromyalgia [18,57].

Several authors have provided theoretical and empirical foundations relevant to these developmental perspectives. For example, Clauw emphasised the roles of altered pain processing, central sensitisation, and stress-system involvement in fibromyalgia [108]. Häuser and colleagues highlighted the multidimensional nature of fibromyalgia, including interactions among biological, psychological, and social factors, as well as the importance of central sensitisation and associated comorbidities [18]. Mayer, Cryan, Dinan, and Bonaz contributed substantially to our understanding of gut–brain interactions, microbiota-related mechanisms, autonomic regulation, vagal pathways, neuroimmune communication, and stress responsiveness [12,13,58,111]. Lastly, broader developmental frameworks have been informed by the work of Bale, Meaney, Gluckman, Barker, and Sandman, particularly regarding prenatal stress programming, epigenetic regulation, foetal programming, developmental plasticity, and the developmental origins of health and disease [56,57,59,109,110].

Within the hypothetical SLEIM framework proposed in this review, prenatal stress-related biological influences affecting ENS-, CNS-, immune-, and microbiota-associated developmental pathways may act as possible contributors to later stress vulnerability [8,9,51,59]. Importantly, this interpretation remains speculative and should be regarded as a hypothesis-generating conceptual model rather than an established mechanism [18,56,57,59,109,110]. Under this framework, altered stress responsivity, reduced adaptive flexibility, and dependency-related coping characteristics may be viewed as potential downstream manifestations of complex developmental, biological, psychological, and environmental interactions [103,104,105,107,112]. However, no direct evidence currently demonstrates that SLEIM causes these characteristics, and alternative explanations—including chronic pain burden, central sensitisation, psychosocial adaptation, and illness-related disability—remain equally plausible.

Accordingly, a more defensible interpretation is that dependency-related characteristics and autonomy-related vulnerabilities may represent possible behavioural manifestations of complex interactions among developmental, biological, psychological, and environmental factors operating across the lifespan. This formulation is more consistent with the available literature on fibromyalgia, stress, DOHaD, and the gut–brain axis while appropriately acknowledging the speculative nature of the proposed developmental framework.

## 8. Discussion

The present hypothesis-driven narrative review explores the possibility that prenatal stress-related biological influences affecting ENS-associated developmental pathways may contribute to later vulnerability to irritable bowel syndrome (IBS), fibromyalgia (FM), and related disorders. The proposed Stress-Induced Long-term Epigenetic Implicit Memory (SLEIM) framework was developed as a theoretical model [1,2] intended to integrate findings from research into prenatal stress, enteric neuroscience, epigenetics, neuroimmune regulation, and the MGBA into a unified conceptual framework.

Importantly, the present review does not propose SLEIM as an established biological mechanism. Rather, it is presented as a hypothesis-generating construct that may help explain how prenatal biological influences could contribute to long-term alterations in physiological responsiveness through interacting ENS-associated, immune, microbial, and CNS pathways. At present, direct empirical evidence supporting the existence of SLEIM as a distinct biological entity remains unavailable, and the proposed framework therefore requires substantial experimental validation.

### 8.1. Prenatal Stress and Parallel Developmental Programming

Accumulating evidence indicates that prenatal stress can influence foetal development through multiple interacting biological pathways, including neuroendocrine [8,9], immune [40,41], metabolic [51,52], microbial [10,53], and epigenetic [8,9,10,53] mechanisms. Contemporary developmental models increasingly recognise that prenatal influences rarely affect a single organ system in isolation [8,56,57]. Rather, the CNS [5,6], ENS [8,30], immune system [51,58], and microbiota-associated pathways [12,53] undergo parallel and interacting developmental processes throughout foetal life.

Maternal stress-related biological signals may influence several developing systems simultaneously through glucocorticoids [8,9,113], inflammatory mediators [8,39], growth factors [51], microRNAs [10,51], extracellular vesicles [55], and microbial metabolites [8,10,39]. Prenatal stress exposure has been associated with alterations in stress regulation [8,9,56], immune function [40,41], neurodevelopment [10,51,52], and later vulnerability to both physical and mental health conditions [57,59,109,110].

Consequently, long-term biological adaptations may emerge through coordinated developmental processes rather than through a single causal pathway. This interpretation is consistent with contemporary concepts of developmental programming and systems biology and is reflected in the conceptual model presented in Figure 1.

### 8.2. IBS and FM Within a Biopsychosocial Framework

The substantial overlap between IBS and FM remains one of the most intriguing observations in contemporary clinical research. Both disorders are characterised by chronic symptom burden [15,82], altered pain processing [74,91], high rates of psychological distress [17,84], sleep disturbance [17,74], fatigue [82,84], autonomic dysfunction [91], and neuroimmune alterations [15,82]. Furthermore, IBS and FM frequently coexist and demonstrate significant overlap in symptomatology [15,16] and quality-of-life impairment [82,90].

Current evidence suggests that neither IBS nor FM can be adequately explained through purely structural pathology [17,71]. Instead, both conditions appear to emerge from complex interactions between biological, psychological, and social factors [103,104]. Central sensitisation [15,82], altered stress responsiveness [74,75], neuroimmune dysregulation [101,102], microbiota-related alterations [84,92], maladaptive illness perceptions [17], coping processes, and psychosocial stressors have all been implicated in their pathophysiology.

The present framework therefore does not view IBS and FM as isolated disorders but rather as multidimensional conditions situated within a broader biopsychosocial context [84,101]. Such an approach may help explain the substantial heterogeneity observed among patients and the considerable variability in symptom presentation, severity, and treatment outcomes [103,104,112].

### 8.3. Potential Role of ENS-Associated Pathways and the Proposed SLEIM Framework

Recent advances in enteric neuroscience have highlighted the remarkable complexity of the ENS [5,6] and its extensive bidirectional interactions with the CNS [27,28], immune system [29], endocrine pathways [29], and intestinal microbiota [29]. The ENS demonstrates considerable neuronal plasticity [5,6], neuroimmune responsiveness, and sensitivity to environmental influences throughout life.

Building upon these observations, the proposed SLEIM framework hypothesises that prenatal stress-related biological influences may become persistently embedded within ENS-associated pathways [1,2] through interacting epigenetic [22,114,115,116], neuroimmune [24,25], neuronal [117,118], glial [14,23], and microbiota-related processes [9,30,100,119]. Epigenetic mechanisms, including DNA methylation [22,114], histone modifications [115,116], and microRNA-mediated regulation [24,25,117,118], have been implicated in long-term biological adaptation and memory-related processes within the CNS [14,23,100,119] and may also contribute to ENS-associated developmental programming [26,54].

However, several important uncertainties remain: the precise biological substrate of SLEIM is unknown; no validated biomarkers currently exist; the mechanisms through which such alterations might be encoded, maintained, or expressed remain speculative; and no direct evidence currently demonstrates that any proposed ENS-associated alterations are independent of CNS developmental processes [1,2]. Consequently, the SLEIM framework should be interpreted as one possible theoretical model among several competing explanations for the observed relationships between prenatal stress, IBS, FM, and stress-related symptomatology.

### 8.4. Dependency-Related Characteristics and Autonomy Vulnerabilities in Fibromyalgia

One objective of the present review was to reconsider behavioural and psychological characteristics frequently reported among individuals living with fibromyalgia and other chronic pain conditions [103,104]. These observations are instead discussed using established constructs derived from chronic pain psychology [106,107], coping research [105], self-regulation theory [105], and adaptation to chronic illness rather than as indicators of personality pathology or developmental deficit [103,104].

A growing body of evidence indicates that many patients with FM experience reduced self-efficacy, increased perceived helplessness, passive or avoidant coping styles, impaired resilience, elevated pain catastrophising, and difficulties maintaining independent self-management in the context of persistent symptoms [104,105,106,107]. These characteristics may reflect complex interactions among chronic pain burden, stress-related processes, illness adaptation, and individual vulnerability factors. The extent to which they represent general consequences of chronic pain, condition-specific characteristics of fibromyalgia, or distinct phenotypic patterns remains to be clarified.

Galvez-Sánchez et al. reported that FM is frequently associated with elevated neuroticism, reduced self-efficacy, psychological distress and maladaptive coping strategies [105]. Similarly, research on IBS suggests that affected individuals often demonstrate lower resilience, lower perceived self-efficacy, greater emotional distress, and increased reliance on maladaptive coping mechanisms compared with healthy controls [99]. Such findings suggest that coping and self-regulatory processes may play an important role across disorders involving chronic pain and gut–brain dysregulation.

Importantly, these characteristics should not be interpreted as personality defects, developmental immaturity, or fixed traits. Rather, they may emerge as understandable adaptations to chronic pain, fatigue, sleep disturbance, functional limitations, repeated healthcare experiences, social invalidation, and long-term illness burden [103,104]. Within the biopsychosocial framework, chronic symptoms and psychological adaptation influence one another dynamically over time.

At the same time, developmental influences may contribute to individual differences in stress responsiveness [8,35], emotional regulation [36,51], coping style [10,52], resilience [8,35], and vulnerability to chronic symptom development [36,51]. Contemporary developmental and stress-vulnerability models suggest that biological programming processes occurring during prenatal and early-life development [59,109] may interact with later environmental experiences [56,110] to shape long-term behavioural and physiological outcomes [57].

Within the proposed SLEIM framework, it may be hypothesised that prenatal stress-related biological influences could interact with later psychological, social, and neurobiological processes [8,35] to contribute to vulnerability profiles characterised by altered stress responsiveness [36,51], maladaptive coping [10,52], increased symptom sensitivity [57,59], and reduced resilience [8,35]. However, these relationships remain speculative and currently lack direct empirical confirmation [36,51]. Future studies are required to determine whether such developmental influences contribute meaningfully to the psychological and behavioural heterogeneity observed among patients with FM and IBS [57,59].

### 8.5. Future Directions

The primary value of the proposed framework lies in its capacity to generate empirically testable hypotheses. Future research should investigate whether prenatal stress exposure is associated with persistent ENS-related biological alterations, characteristic epigenetic signatures, neuroimmune changes, microbiota-related biomarkers, autonomic dysregulation, or identifiable clinical phenotypes later in life.

In particular, prospective longitudinal birth-cohort studies integrating maternal stress assessments, microbiome profiling, epigenetic analyses, neurodevelopmental measures, and long-term clinical outcomes would be particularly informative. Such approaches could help clarify whether prenatal stress-related biological influences contribute to later vulnerability to IBS, FM, or related disorders.

Future mechanistic investigations should also examine potential interactions among enteric neurons, enteric glial cells, immune pathways, microbial metabolites, and stress-response systems [9,40]. Experimental animal models may provide opportunities to evaluate whether prenatal stress can induce persistent ENS-associated alterations [51,55] and whether such changes influence later physiological responsiveness [9,40].

Studies capable of distinguishing ENS-associated developmental influences from alternative CNS-centred explanations will be equally important. Future work should therefore compare competing developmental models, including central sensitisation frameworks, neuroimmune models, stress-vulnerability models, and MGBA models.

Ultimately, the relationships between prenatal stress, developmental programming, the MGBA, IBS, FM, and chronic pain remain incompletely understood. The proposed SLEIM framework represents one possible conceptual approach for organising these observations and generating new research questions, but whether such a framework reflects an underlying biological reality remains to be determined through future empirical investigation.

## 9. Limitations

The present work is a hypothesis-driven narrative review and theoretical synthesis rather than an experimental or clinical study. Several important limitations should therefore be acknowledged.

First, the proposed Stress-Induced Long-term Epigenetic Implicit Memory (SLEIM) framework remains a theoretical construct. Although individual components of the model—including prenatal stress, epigenetic regulation, enteric neurobiology, MGBA signalling, neuroimmune interactions, and chronic pain mechanisms—are supported by varying degrees of empirical evidence, direct evidence demonstrating the existence of SLEIM as a distinct biological entity is currently lacking.

Second, no specific biomarkers, physiological signatures, imaging findings, or experimental measures currently exist that can directly identify, quantify, or verify SLEIM-related processes in humans. Consequently, the proposed framework should be viewed primarily as a hypothesis-generating model intended to stimulate future empirical investigation.

Third, the biological pathways linking prenatal stress, ENS development, microbiota-associated processes, chronic pain, IBS, and FM are highly complex and involve multiple interacting systems. Alternative explanations—including central sensitisation, neurodevelopmental models, neuroimmune mechanisms, autonomic dysregulation, genetic susceptibility, environmental influences, psychosocial factors, and illness-related adaptation—may explain many of the observed clinical phenomena discussed in this review.

Fourth, although IBS and FM frequently coexist and share several biological and clinical characteristics [15,82], the present review does not establish a causal relationship between these disorders [16,90]. The temporal sequence and mechanistic pathways linking IBS and FM remain incompletely understood [95,120] and require further investigation [83,121].

Fifth, the psychological characteristics discussed in relation to FM and IBS should not be interpreted as universal, disease-specific, or deterministic features. Constructs such as reduced self-efficacy, passive coping, avoidance, resilience deficits, dependency-related characteristics, or autonomy vulnerabilities may emerge through complex interactions among chronic symptoms, developmental influences, psychosocial experiences, personality factors, and long-term adaptation to illness.

Finally, the proposed developmental framework remains speculative because prospective longitudinal studies directly examining prenatal stress exposure, ENS development, microbiota-related processes, and later vulnerability to IBS and FM are currently limited. Future multidisciplinary research integrating developmental neuroscience, neurogastroenterology, microbiome science, epigenetics, immunology, and chronic pain research will be necessary to evaluate the validity of the proposed model.

## 10. Conclusions

Prenatal stress is increasingly recognised as an important developmental influence capable of affecting multiple biological systems [8,35] through interacting neuroendocrine [36,39], immune [9,40], microbial [41,43], and epigenetic pathways [42,44,45,122]. Contemporary evidence suggests that foetal development involves complex and interconnected processes affecting the CNS [37,38], ENS [46,47], immune system [48,49], and microbiota-associated pathways simultaneously [10,50,51,52]. These observations provide a biological foundation for investigating how early-life influences may contribute to later vulnerability to disorders involving gut–brain interactions and chronic pain [56,57,59,109,110].

Fibromyalgia and irritable bowel syndrome demonstrate substantial overlap across clinical, neurobiological, psychological, and microbiota-associated domains [15,82]. Their frequent coexistence [16,74], together with evidence implicating altered pain processing [75,101], stress-system dysregulation [79,102], neuroimmune mechanisms [90,91], and MGBA disturbances [84,92], suggests that partially shared biological pathways may contribute to both conditions [17,95]. However, the precise nature of these relationships remains incompletely understood.

The Stress-Induced Long-term Epigenetic Implicit Memory (SLEIM) framework proposed in this review represents a hypothesis-generating conceptual model intended to stimulate further investigation into the potential developmental consequences of prenatal stress. The model does not constitute an established biological mechanism and currently lacks direct empirical validation. Nevertheless, it offers a theoretical framework [1,2] through which findings from developmental neuroscience, enteric neurobiology, microbiome research, epigenetics, neuroimmunology, and chronic pain research may be integrated and examined within a common perspective.

Future multidisciplinary research will be required to determine whether prenatal stress-related biological influences contribute to long-term alterations in ENS-associated pathways and whether such alterations are relevant to later vulnerability to IBS, FM, or related disorders. Clarifying these relationships may ultimately improve our understanding of developmental contributions to chronic pain and disorders of gut–brain interaction.

## Figures and Tables

**Table 1:** Principal domains of overlap between irritable bowel syndrome (IBS) and fibromyalgia (FM) and their relevance to the proposed conceptual framework.

Domain	Evidence in IBS	Evidence in FM	Level of Evidence	Key References
Chronic pain and sensory amplification	Visceral hypersensitivity is a core feature of IBS and contributes to symptom generation	Widespread pain, pain amplification, and central sensitivity are characteristic features of FM	Strong	[17,71,72,73,92,93]
CNS alterations	Altered pain processing, emotional regulation, and functional brain connectivity have been reported	Central sensitisation and altered CNS pain processing are consistently reported	Strong	[17,18,93]
MGBA involvement	Dysbiosis, altered microbial metabolites, gut barrier dysfunction, and microbiota-related gut–brain signalling have been described	Altered microbiota composition, metabolomic abnormalities, and microbiota-associated pain-related changes have been reported	Moderate	[11,12,13,15,71,73,91,96,97]
Neuroimmune interactions	Low-grade inflammation, immune activation, and altered gut barrier-related immune signalling have been described	Immune and inflammatory abnormalities have been proposed or reported	Moderate	[15,18,73,84,92]
Stress-system dysregulation	Altered stress responsiveness, and psychological stress-related symptom exacerbation are frequently reported	Stress-system abnormalities, altered stress responsiveness, and stress-related symptom persistence are frequently observed	Moderate	[17,18,71,73,74,79,92]
Psychological comorbidity	Anxiety, depression, psychological distress, and maladaptive coping are common in IBS	Anxiety, depression, sleep disturbance, psychological distress, and maladaptive coping are highly prevalent in FM	Strong	[59,74,81,85,98]
Autonomic nervous system dysfunction	Altered autonomic regulation and vagal/autonomic involvement have been reported in IBS and gut–brain interaction disorders	Autonomic dysfunction has been reported in FM and overlapping central sensitivity conditions	Moderate	[13,18,19,99]
Epigenetic regulation	Genetic and epigenetic mechanisms have been proposed as contributors to IBS vulnerability and gut–brain interaction mechanisms	Genetic and epigenetic mechanisms have been proposed in FM pathophysiology	Emerging	[1,2,14,22,23,26,54,86,88,100]
Familial and genetic susceptibility	Genetic contributions have been proposed in IBS pathophysiology	Familial aggregation and heritability are well documented in FM	Moderate	[71,86,88,89]
Relationship with environmental stressors	Stress is associated with symptom exacerbation and disease vulnerability in IBS	Stress is associated with symptom severity, persistence, and vulnerability in FM	Strong	[17,18,74,75,80,92,101,102]
Developmental influences	Prenatal, early-life, and developmental influences are increasingly discussed in relation to gut–brain axis vulnerability	Prenatal, developmental, and stress-vulnerability factors are compatible with broader developmental models of chronic pain vulnerability, although FM-specific causal evidence remains limited	Emerging	[1,2,8,9,10,30,34,36,40,41,51,52]
SLEIM-related developmental framework	Hypothetical contribution through ENS-associated developmental programming	Hypothetical contribution through ENS-associated developmental programming	Hypothesis-generating	[1,2,94]

Abbreviations: IBS, irritable bowel syndrome; FM, fibromyalgia; CNS, central nervous system; ENS, enteric nervous system; SLEIM, Stress-Induced Long-term Epigenetic Implicit Memory. Note: The purpose of this table is not to imply a common aetiology but rather to summarise the principal domains in which IBS and FM demonstrate overlapping clinical, neurobiological, psychological, and developmental characteristics according to current evidence. The SLEIM framework is presented as a hypothesis-generating theoretical construct and not as an established biological mechanism.

## Data Availability

No new data were created or analyzed in this study. Data sharing is not applicable to this review article.
